# Costs of breast cancer care in Mexico: analysis of two insurance coverage scenarios

**DOI:** 10.3332/ecancer.2015.587

**Published:** 2015-10-28

**Authors:** María Cecilia González-Robledo, Rebeca Wong, Héctor Arreola Ornelas, Felicia Marie Knaul

**Affiliations:** 1 Center for Research in Health Systems, National Institute of Public Health, Av. Universidad 655 cerr. Los Pinos y Caminera, Col. Santa María Ahuacatitlán C.P. 62100, Cuernavaca, Morelos, Mexico; 2 WHO Collaborating Centre/PAHO Aging and Health, University of Texas Medical Branch, Texas, USA; 3 Economic Research and Research Promotion Council of Competitiveness and Health, Mexican Health Foundation, Mexico; 4 Miami Institute of Americas & Miller School of Medicine, Miami University, Miami, USA; Mexican Health Foundation & Founding President of Tómatelo a Pecho A.C., México; * At the time of writing the paper, Director of the Harvard Global Equity Initiative at Harvard University

**Keywords:** breast neoplasms, direct service costs, health financing, Mexico

## Abstract

**Background:**

Breast cancer (BC) is a major cause of disease and death worldwide. In addition to its contribution to mortality and disability, it is a major economic burden both public and private.

**Objective:**

To estimate the average direct medical cost/year of care for the diagnosis and treatment of BC in two coverage scenarios in Mexico: What is ‘ideal’ based on service usage patterns according to international guidelines and what is ‘current’ using the service usage patterns of suppliers in Mexico.

**Material and Methods:**

The pattern and intensity of use of procedures for the care of BC in the Mexican Social Security Institute (IMSS) for 2009 were identified and prices were associated using the guidelines from the System of Social Protection in Health (SPSS) and the IMSS for the current scenario and the ideal scenario, international patterns (Breast Health Global Initiative BHGI after its acronym in English) were used and prices were associated from the SPSS guidelines.

**Results:**

The annual average direct medical cost per patient in the ‘current’ scenario was 8557 US$, while the cost in the ‘ideal’ scenario was 4554 US$. There are differences in costs between ‘what we do’ and ‘what should be done’, due to differences in the implementation of the interventions for the treatment of the different stages of the disease. A proportional increase in the average cost was also identified as the diagnosis stage advanced (from I to III).

**Conclusions:**

Given that in Mexico there is universal insurance coverage for the treatment of BC, it is necessary to use economic resources more efficiently. It is necessary to continue to examine this topic in more depth and the next step will be to assess the effectiveness of both scenarios in order to provide enough evidence for the decision-making process.

## Introduction

Breast cancer (BC) is a major cause of disease and death in the world. GLOBOCAN data reported in 2012 indicates that it is the most common cancer among women in both developed and developing countries and indicates that 1.67 million new cases were diagnosed in the study period. The incidence is highest in countries of Western Europe with a rate of 96 per 100,000 women, while in African countries, the rate is one of the lowest with 27 per 100,000. However, it is the most frequent cause of cancer death in women in the less developed regions (324,000 deaths, 14.3% of the total). Mortality rates are lower than those of incidence due to the fact that survival is more favourable in the more developed regions [1–3].

In Mexico, BC is the second leading cause of cancer death in women older than 35 years. Incidence and mortality rates have shown an upward trend over time (between 1980 and 2012) in this population. Data reported by GLOBOCAN 2012 indicates that the incidence rate for BC in the country is 35.4 and the mortality rate is 9.7 per 100 thousand women. Of the confirmed cases, slightly more than 50% were diagnosed in advanced stages (III and IV), which substantially decreases the likelihood of survival for 5 years, even in spite of receiving treatment (Ministry of Health, 2007) [4].

European studies estimated that the costs of BC are of significant magnitude and variability. In Sweden (2002), the average annual cost per patient for BC care was 13,238 US$ and in France (2004) 36,073 US$, which included the assessment of both direct and indirect costs [5, 6]. In Latin America and the Caribbean, the economic burden of BC has not been studied much. In Brazil, there were two studies in 2009, one in the public and another in the private sector. The results show differential costs due to the fact that each sector follows different treatment alternatives. The average annual cost per patient for the private sector was 15,426 US$ compared to 4,757 US$ for the public sector [7, 8]. In Mexico, Knaul *et al*, conducted a study in Mexico in a social security institution with data from 2006, estimating the average cost per year per patient to be 6734 US$ [9]. On the other hand, data published in 2010, in the country, indicate that in the public health system, the treatment of breast cancer accounted for 21.2% of the total expenditure allocated by the fund for the protection against catastrophic expenses (FPGC) [10], which in turn represented 1.98% of the total public expenditure on health in the country.

From a social perspective, BC is a disease that in addition to contributing to mortality and disability, adds a major public and private economic burden (this last generator of out-of-pocket spending is due to processes of care which are not included within the care package or indirect costs that could result in impoverishing costs for households with fewer economic resources) [5, 6, 11]. In the Mexican case, the economic burden is taken up to a significant degree by the public health sector due to the fact that all women have the right to comprehensive care through social security or public insurance provided by the System of Social Protection in Health (SPSS) called Seguro Popular (SP) [12]. In view of this wide coverage, a better understanding of the treatment costs of the BC is an important input to the planning of health resources.

This article presents an estimate of the direct medical costs of diagnosis and treatment of patient with BC/year in the public health sector of Mexico, considering two scenarios: ‘current’ and ‘ideal’, which will be explained in the materials and methods section. The inclusion of the two scenarios is intended to show the cost differences obtained by the use of differential patterns between the procedures currently used in Mexico’s health sector and those defined by international recommendations, in order to provide information for decision makers to carry out the planning of financial resources that will be needed in the future to address this health problem. It is hoped that this information will help people understand and anticipate the future economic burden for the Mexican health care system.

## Materials and methods

The medical procedures included in the estimate of the cost of diagnosis were medical consultations (contained within the procedures), biopsies (by aspiration or surgery), radiology studies (mammography, ultrasound), clinical laboratory studies (CBC and others), pathology studies, and other diagnostic studies (bone densitometry). The procedures for the treatment were built by stage or step of diagnosis and included the following: surgical interventions (radical and conservative surgery), chemotherapy (different schemes), radiotherapy, and hormone therapy (oestrogen inhibitors, HER2).

The cost estimate was conducted in the following three phases:

- Phase I: Determination of the cost of each medical procedure/service in a year per patient and stage of the disease (I to IV).
  $$Cp_{j}=Q_{j}\times P_{j}$$
  Where *C*p_j_ is cost of the procedure, *Q*_j_ is quantity used in the procedure (on average per patient), *P*_j_ is the price of the procedure and j is the procedure.
- Phase II: Determination of the direct medical cost by intervention (diagnosis, surgery, chemotherapy, and radiotherapy) per patient year and stage of the disease.
  $${\mathrm{CI}}_{k}=\Sigma Cp_{j}$$
  Where *CI*_k_ is the cost of the intervention, j is the interventions for the diagnosis and/treatment: surgery, chemotherapy, radiation therapy, and k is the stages of diagnosis (I to IV).
- Phase III: Determination of the total direct medical cost per patient/year, taking into account the diagnostic steps and proposed scenarios: (a) of universal coverage and (b) ideal.
  $$C_{\mathrm{total}}=\Sigma {\mathrm{CI}}_{k}\;\times \;{\mathrm{NM}}_{k}$$
  Where *NM*_k_ is the number of women at every stage of diagnosis (I to IV), k is the stages of diagnosis (I to IV), and j is the interventions for the diagnosis and/ treatment: surgery, chemotherapy, radiation therapy.

A descriptive cross-sectional study was conducted in 2009. The unit of analysis were women aged 25 and over, diagnosed and treated in the Instituto Mexicano de Seguro Social (IMSS) using prevalence data in all stages of diagnosis.

It had various sources of information. Reference was made to the International Classification of Diseases, ICD-10 [13], code C50 and steps determined by the Treatment Guidelines for Patients (GTP) of the American Cancer Society VIII version and the Norma Oficial Mexicana (2002) [14, 15]; therefore, stages I, II (early) and III, IV (late stage) were taken [16]. These sources were used for the ‘current’ scenario. For the ‘ideal; scenario, the interventions suggested by the International Guidelines developed by the World Initiative for Breast Health were taken into account, the latter being stratified according to the level of resources that a country has. For this particular case, a ‘broad level’ was taken [17]. To estimate the services usage pattern, the Operational Medical Medical Information System IMSS was used, in particular the subsystems SUI-7 and SUI-13, which correspond to outpatient and hospital costs, respectively. This pattern of service use was revised and adjusted for the year 2009 [18, 19]. The estimates of the direct medical costs of care were based on the tabulators of the Ministry of Health (Official Gazette of the Federation), the tabulator for recovery quotas of the INCAN and the tabulator of the IMSS, estimated all costs at values of 2009 [20, 21]. The data analysis was carried out using Microsoft Excel®.

Two models were used to estimate direct medical costs: (1) The usage pattern of services was estimated according to a non-linear regressive chain model developed by Knaul Markov, Arreola *et al* (2009) through a transition matrix of the disease that meant a progressive and substantial step forward from one stage to another without allowing improvement in previous states [9], and (2) the cost estimate based on the model adapted by Brown and Yabroff [22, 23] that includes direct medical costs paid by third parties. The selection of this segment of the model is justified because it is one of the components of greatest interest among decision-makers for the establishing of public financial amounts which are required for the care of BC in the population, which, according to the published literature, can represent up to 55% of the total costs of care [5, 24].

Costs were estimated under the assumption that the pattern of use of procedures and services for the IMSS was the same as in all other social security institutions in Mexico: ISSSTE, PEMEX, SEDENA, SEMAR and for those who are receiving care from SPSS. The reason for this is that IMSS information records had a unique identification code that allowed staff to follow cases, while the other social security institutions and the care records of the SPSS did not; therefore, keeping track of each case (each diagnosed woman) was not possible. However, given that there are consensual guidelines for the management of these patients throughout the country, it was possible to work under this assumption. Treatment costs were calculated based on the diagnostic stage, as is recommended by the literature on the subject, given that the stage of the disease can directly influence the costs of care [23–25].

The following scenarios were handled for estimating the costs of medical care:

1. *Current Scenario*. For the cost estimate, the usage pattern of services of the IMSS and the tabulators of prices of services of the IMSS and SPSS is employed.
2. *Ideal Scenario*. Constructed with the usage patterns of the procedures and services identified by the guidelines from the Breast Health Global Initiative (BHGI because of its acronym in English) in the level of comprehensive resources. The difference with the above scenario is the type and amount of services and procedures used. The tabulator of prices used for estimating costs was that of the SPSS [17].

The project was approved by the commissions of inquiry, ethics, and biosafety of the INSP.

We have defined the broad level as both the public services, such as the social security (IMSS), offer treatments consistent with the procedures described for this level by the international guidelines. This scenario was chosen because the importance of international regulations for breast health care based on evidence has been globally recognised, which improves in quantifiable terms the outcomes of BC, to achieve the best standard of care that would be practical in each environment [17].

## Results

The group with the highest number of diagnosed BC cases was women aged between 45 and 64 years with 53.5%. This was followed by those aged between 25 and 44 years with 28% and those aged 65 and over with 18.5%. About 46.2% of cases were diagnosed in the early stages (I and II).

Table 1 shows the direct medical costs per procedure/year in the care of BC patients in 2009. It is important to explain that this table shows the costs differentiated by type of insurer: Mexican Social Security Institute (IMSS) and Public Health Insurance (SPSS) [sic]; this is due to the tabulators of prices being different. Nevertheless, the overall results show the average of both estimates.

The average direct medical cost/year of care in the public sector for a woman was US$ 8557. The average direct medical cost/year of diagnosis was US$ 303. The average treatment costs: surgery US$ 1163, radiotherapy US$ 376, and chemotherapy US$ 6735. These costs were analysed by stages of diagnosis. The results were as follows: stage I US$ 6500, stage II US$ 9981, stage III US$ 12,757, and stage IV US$ 5069 (palliative care costs are not included).

The highest costs are concentrated in chemotherapy procedures (87%) followed by surgical procedures (10%), independent of stage of diagnosis. The most frequently used types of chemotherapy for the first stages (I and II) were FEC (5-fluorouracil (5FU), epirubicin, and cyclophosphamide) and CMF (cyclophosphamide, methotrexate, and 5FU) and for the final stages, FEC, epirubicin, and taxotere (III), and capecitabine and navelbine (IV). The most common surgical procedures performed were lumpectomy and sentinel lymph node biopsy (I and II), and mastectomy and lymph node dissection (III and IV).

‘Ideal’ scenario costs are shown in Table 2.

The average direct medical cost/year for a female patient with the pattern of use of BHGI guidelines will be US$ 4554. Treatment costs differentiated by stage of diagnosis will be stages I and II US$ 3368, stage III US$ 5995, and stage IV US$ 5484 (without palliative care). Costs differentiated by the type of intervention will be diagnosis US$ 774, surgical procedure US$ 722, chemotherapy US$ 11,698, and radiotherapy US$ 9991.

A comparison of costs between the proposed scenarios was performed yielding interesting results, lower than those established in the pattern of use of international guidelines. This difference will be analysed in the discussion. There are two types of differences, namely quantitative and qualitative. A comprehensive package of services can be seen in the public service sector of IMSS ranging from conservative to radical surgery (10 different types of procedures) and numerous and extensive chemotherapy protocols (5–7), as opposed to the international recommendations of a more restricted pair of surgical models and chemotherapy protocols (4 in total) as indicated by first generation international guidelines, even at the level of comprehensive resources.

## Discussion

National cost estimates for the care of BC is a complicated process due to the nature of the information, the different levels of data aggregation, limited access to information systems, as well as problems of timeliness and coverage of the information.

Several aspects of the results of this study are consistent with the data published in literature. Firstly, the majority of women are diagnosed in the advanced stages of the disease. However, if we compare these figures with those reported by SPSS in 2007, we can see an upward trend in the early detection of lesions which could indicate that the national screening programs being conducted are successfully achieving their objectives [26, 27].

Another aspect that coincides with the published literature is that the majority of detected BC cases occur in patients aged under 65 [28]. This study observed that 78% of the population covered by IMSS and 85% of those covered by SPSS are diagnosed women aged under 64. This causes important productivity losses due to disability and premature mortality caused by the disease (aspects that are not covered by this study), as well as the consequences on the quality of life for these women [5].

With regards to the average direct medical care costs/year, differences were observed between the service providers (paying agents) and studied scenarios. The service providers originate from the tabulators of prices, considering that the assumed pattern and intensity of use of procedures were the same. The highest costs are concentrated in the population covered by IMSS (annual average cost of US$ 10,071 as opposed to US$ 7036 with SPSS). Several explanatory hypotheses can be discussed; for example, SPSS may have a better ability to negotiate prices in the purchase of medicines or the management of financial resources in both institutions is different which could elevate IMSS costs. Nevertheless, the objective of this study was never to understand the reason for these differences between the service providers; thus, in the future, it is recommended to study who has the highest rate of effectiveness and efficiency with a view to making the best use of resources.

Upon comparing the ‘ideal’ and ‘current’ scenarios that differ in the pattern and intensity of use of procedures and in the tabulators of prices, an important difference was seen between the costs of the first and second scenarios. To make it more comparable, the estimated average with the SPSS tabulators was taken as the current scenario cost, which was US$ 7036, and the ideal scenario cost, which was US$ 4554; this shows that the latter would still be lower than the cost of the current scenario by at least 35%. This could also lead to applying the BGHI’s recommended procedures in Mexico, which could provide a scenario of greater equity of care for women nationwide, homogenising the provision of services. Consequently, it is necessary to perform studies that allow for a deeper understanding of the efficiency of both scenarios in order to make an informed decision.

Another important discovery is that this study established that the average direct medical costs/year in diagnosis and treatment relate to 45% of the total expenditure of the fund for the protection against catastrophic expenses (FPGC) in the current scenario (SPSS) when analysed with figures reported by SPSS [29].

An important point for future study will be to determine what benefits a country like Mexico (middle income) could bring when adopting the international guidelines within the SPSS package of services, in terms of effectiveness.

Two of the main constraints of this study were: firstly, an underestimation of care costs; only costs attributable to the diagnosis and treatment of BC stages were valued in a year, leaving aside costs for follow-up, monitoring and palliative care and, furthermore, indirect costs were not estimated as recommended by the available literature. Nevertheless, the choice of these stages is justified on the premise that fewer than 50% of care costs are focused on them (direct medical costs) as recommended by the available literature. The generated information is therefore considered useful for decision-makers in charge of the financial planning of the health system.

Secondly, the pattern of use of procedures and services can have biased information as it was assumed that all Mexican institutions presented the same pattern of use of IMSS, due to SPSS information systems not being allowed to do a follow-up on a cohort within a specified time frame due to the lack of a unique identifier. Furthermore, there is no access to the information of other social security institutions (ISSSTE, PEMEX, SEDENA, SEMAR). Nonetheless, it is hoped that this situation will not represent a problem as the country relies on consensual protocols and management guidelines that somehow unify these patterns of use [30].

## Conflict of interest

The authors state that there are no financial, labour or other relations that pose as a conflict of interest regarding this study. That is, we have not received any ‘benefits in property, hospitality, or subsidies’; however we acknowledge the financial support of Sanofi US for the field work and the results of this research have no particular interest in the investigation.

## Conclusion

The estimated direct medical costs paid by third parties at the national level is important for budgetary planning of care of CM, even more, considering that universal insurance coverage for care exists in Mexico. We still need to understand the reasons for the differences in costs related to the scenarios, but we believe we have taken the first step.

## Figures and Tables

**Table 1.** Direct medical costs per procedure/year by stage of diagnosis of BC. Current scenario in Mexico 2009.

	Resource or service	Use of service^*^	Unit Cost	
			IMSS	SPSS
**Diagnosis**				
	Complete blood count (CBC)	1.00	$54.14	$34.87
	Fine-needle aspiration biopsy	0.29	$459.00	$832.00
	Open biopsy	0.67	$8765.00	$2963.86
	Bilateral mammogram	1.00	$213.00	$1484.00
	Liver function tests (LFTs)	1.00	$245.00	$157.78
	Electrocardiography (ECG)	1.00	$356.00	$229.26
	Monitoring of bones	1.00	$367.00	$236.35
	*Subtotal*		$10,459.10	$5938.12
**Stage-I treatment**	*Surgical interventions*	0.98	$16,791.61	$10,813.80
	• Lymph node dissection	0.04	$13,713.26	$8831.34
	• Lumpectomy + lymph node dissection	0.15	$13,538.89	$8719.05
	• Lumpectomy + lymph node dissection + sentinel lymph node biopsy	0.74	$16,415.65	$10,571.68
	• Mastectomy + sentinel lymph node biopsy	0.02	$23,603.19	$15,200.46
	• Mastectomy + lymph node dissection	1.0	$13,736.19	$8846.11
	• Reconstructive surgery	0.01	$29,559.32	$19,036.20
	• Mastectomy + reconstructive surgery	0.02	$30,952.28	$19,933.27
	• Mastectomy + sentinel lymph node biopsy + lymph node dissection + reconstructive surgery	0.02	$41,456.47	$26,697.97
	*Radiotherapy*	0.54	$11,759.82	$8,231.87
	*Chemotherapy*	0.69	$75,224.26	$49,648.01
	FEC (6–8 cycles)	0.20	$171,913.65	$113,463.01
	FEC (4 cycles)	0.18	$99,217.57	$65,483.60
	CMF (6 cycles)	0.19	$6541.20	$4317.19
	Epirubicin + Taxotere (4 cycles)	0.02	$124,198.00	$81,970.68
	Trastuzumab (Weekly for 8 months)/2	0.08	$248,257.21	$163,849.76
**Stage-II treatment**	*Surgical interventions*	0.96	$18,003.15	$11,594.03
	• Lymph node dissection	0.04	$13,713.26	$8831.34
	• Lumpectomy + lymph node dissection	0.24	$13,538.89	$8,719.05
	• Lumpectomy + lymph node dissection + sentinel lymph node biopsy	0.48	$16,415.65	$10,571.68
	• Mastectomy + sentinel lymph node biopsy	0.03	$23,603.19	$15,200.46
	• Mastectomy + lymph node dissection	0.03	$13,736.19	$8846.11
	• Mastectomy + sentinel lymph node biopsy + lymph node dissection	0.10	$24,115.00	$15,530.06
	• Reconstructive surgery	0.02	$29,559.32	$19,036.20
	• Mastectomy + reconstructive surgery	0.02	$30,952.28	$19,933.27
	• Mastectomy + sentinel lymph node biopsy + lymph node dissection + reconstructive surgery	0.04	$41,456.47	$26,697.97
	*Radiotherapy*	0.38	$11,759.82	$5792.18
	*Chemotherapy*	1.18	$132,983.07	$87,768.83
	FEC (6–8 cycles)	0.35	$171,913.65	$113,463.01
	FEC (4 cycles)	0.21	$99,217.57	$65,483.60
	CMF (6 cycles)	0.27	$6541.20	$4317.19
	GemCarbo (6 cycles)	0.02	$57,509.40	$37,956.20
	Capecitabine (6 cycles)	0.01	$67,192.80	$44,347.25
	Navelbine (6 cycles)	0.01	$38,347.80	$25,309.55
	Epirubicin + Taxotere (4 cycles)	0.12	$124,198.00	$81,970.68
	Trastuzumab (Weekly for 8 months)/2	0.13	$248,257.21	$163,849.76
**Stage-III treatment**	*Surgical interventions*	0.96	$22,203.78	$14,299.23
	• Lumpectomy + lymph node dissection	0.01	$13,538.89	$8,719.05
	• Lumpectomy + lymph node dissection + sentinel lymph node biopsy	0.02	$16,415.65	$10,571.68
	• Mastectomy + sentinel lymph node biopsy	0.09	$23,603.19	$15,200.46
	• Mastectomy + lymph node dissection	0.26	$13,736.19	$8846.11
	• Mastectomy + sentinel lymph node biopsy + lymph node dissection	0.41	$24,115.00	$15,530.06
	• Reconstructive surgery	0.04	$29,559.32	$19,036.20
	• Mastectomy + reconstructive surgery	0.06	$30,952.28	$19,933.27
	• Mastectomy + sentinel lymph node biopsy + lymph node dissection + reconstructive surgery	0.08	$41,456.47	$26,697.97
	*Radiotherapy*	0.12	$11,759.82	$8,231.87
	*Chemotherapy*	1.43	$177,968.02	$117,458.90
	FEC (6–8 cycles)	0.25	$171,913.65	$113,463.01
	FEC (4 cycles)	0.14	$99,217.57	$65,483.60
	CMF (6 cycles)	0.18	$6541.20	$4317.19
	GemCarbo (6 cycles)	0.09	$57,509.40	$37,956.20
	Capecitabine (6 cycles)	0.05	$67,192.80	$44,347.25
	Navelbine (6 cycles)	0.06	$38,347.80	$25,309.55
	Epirubicin + Taxotere (4 cycles)	0.44	$124,198.00	$81,970.68
	Trastuzumab (Weekly for 8 months)/2	0.16	$248,257.21	$163,849.76
**Stage-IV treatment**	*Surgical interventions*	1.00	$24,594.50	$15,838.86
	• Lymph node dissection	0.04	$13,713.26	$8831.34
	• Mastectomy + sentinel lymph node biopsy	0.15	$23,603.19	$15,200.46
	• Mastectomy + lymph node dissection	0.21	$13,736.19	$8846.11
	• Mastectomy + sentinel lymph node biopsy + lymph node dissection	0.32	$24,115.00	$15,530.06
	• Reconstructive surgery	0.05	$29,559.32	$19,036.20
	• Mastectomy + reconstructive surgery	0.09	$30,952.28	$19,933.27
	• Mastectomy + sentinel lymph node biopsy + lymph node dissection + reconstructive surgery	0.14	$41,456.47	$26,697.97
	*Radiotherapy*	0.04	$11,759.82	$8,231.87
	*Chemotherapy*	0.48	$52,296.54	$34,515.71
	FEC (6–8 cycles)	0.01	$171,913.65	$113,463.01
	FEC (4 cycles)	0.02	$99,217.57	$65,483.60
	CMF (6 cycles)	0.04	$6541.20	$4317.19
	GemCarbo (6 cycles)	0.05	$57,509.40	$37,956.20
	Capecitabine (6 cycles)	0.13	$67,192.80	$44,347.25
	Navelbine (6 cycles)	0.14	$38,347.80	$25,309.55
	Epirubicin + Taxotere (4 cycles)	0.03	$124,198.00	$81,970.68
	Trastuzumab (Weekly for 8 months)/2	0.05	$248,257.21	$163,849.76
	Bevacizumab	0.04	$380,581.01	$251,183.47

* The ‘Use of service’ column refers to the number of times a procedure is performed by a user in one-year follow-up.

**Sources:** Data of the IMSS and FPGC Operative Medical Information System (SIMO). Tabulator for recovery quotas of the INCAN. Unit costs for medical care by level of care. Undersecretary of Income. Unit for Income Policy. Mexican Secretariat of Finance and Public Credit. 2009. IMSS 2009 published in DOF (6-03-2009 second section 65–68).

**Table 2.** Direct medical costs per procedure/year by stage of diagnosis of BC. Ideal scenario in 2009.

Resource or service		No. of times of service	Unit cost
**Diagnosis**	Analysis of complete blood chemistry	1.00	$34.87
	Percutaneous needle biopsy	0.29	$832.00
	Surgical biopsy	0.67	$2963.86
	Diagnostic mammogram	1.00	$1484.00
	Basic chest x-ray	1.00	$1064.00
	Liver ultrasound	1.00	$157.00
	Bone scintigraphy	1.00	$236.34
	Subtotal	–	$6772.07
**Stage-I treatment**	*Surgical interventions*	1.00	$8846.11
	Mastectomy + lymph node dissection	1.00	$8846.11
	*Radiotherapy*	1.00	$16,381.68
	*Chemotherapy*	1.00	$28,895.37
	AC (4 cycles) + taxanes	0.61	$81,970.68
	CMF (6 cycles)	0.27	$4,317.19
	Traztuzumab (for HER2-positive patients)	0.12	$163,849.76
**Stage-II treatment**	*Surgical interventions*	1.00	$8846.11
	Mastectomy + lymph node dissection	1.00	$8846.11
	*Radiotherapy*	1.00	$16,381.68
	*Chemotherapy*	1.00	$28,895.37
	AC (4 cycles)	0.65	$81,970.68
	CMF (6 cycles)	0.23	$4317.19
	Traztuzumab (for HER2-positive patients)	0.12	$163,849.76
**Stage-III treatment**	*Surgical interventions*	1.00	$9931.34
	• Mastectomy + lymph node dissection	0.39	$15,200.46
	• Mastectomy + sentinel lymph node biopsy	0.61	$8846.11
	*Radiotherapy*	1.00	$16,381.68
	*Chemotherapy*	1.00	$54,308.12
	CMF (6 cycles)	0.13	$4,317.19
	AC (4 cycles)	0.46	$81,970.68
	EC	0.13	$81,970.68
	FAC	0.17	$65,483.60
	Traztuzumab (for HER2-positive patients)	0.11	$163,849.76
**Stage-IV treatment**	*Surgical interventions*	1.00	$11,387.85
	• Mastectomy + sentinel lymph node biopsy	0.40	$15,200.46
	• Mastectomy + lymph node dissection	0.60	$8846.11
	*Radiotherapy*	1.00	$16,381.68
	*Chemotherapy*	1.00	$45,933.50
	CMF (6 cycles)	0.09	$4317.19
	AC (4 cycles)	0.11	$81,970.68
	Capecitabine	0.31	$44,347.25
	Navelbine	0.35	$25,309.55
	Trastuzumab	0.14	$163,849.76

**Sources**: BC diagnosis and treatment BHGI 2007 guidelines (broad level). Tabulator for recovery quotas of the INCAN. Unit costs for medical care by level of care. Undersecretary of Income. Unit for Income Policy. Mexican Secretariat of Finance and Public Credit. 2009.
